# Supplementary material for: Biphasic acclimation for simultaneous wide-field fluorescent Ca2+ imaging and fMRI of awake mice
Source: Imaging Neurosci (Camb). 2026 Sep 10;4:IMAG.a.1357. doi: 10.1162/IMAG.a.1357 (PMC13563530; doi:10.1162/IMAG.a.1357)
Supplement: Supplementary file 1 [file IMAG.a.1357_supp.pdf]

Supplementary material

Supplementary Tables

Table S1. Animal cohort breakdown, all data used in analyses

	**Age** (m)	**Longitudinal *** (N = animal, n = runs)	**Cross-sectional**	**State**
**iso_4m**	4m	N = 5/4+2** m/f, n = 27	N.A.	Anesthesia
**iso_6m**	6m	N = 5/4 m/f, n = 27	N.A.	Anesthesia
**iso_9m**	9m	N = 0/1ⵜ+1** m/f, n = 3	N = 4/2 m/f, n = 18	Anesthesia
**awake_9m**	9m	N = 3/2 m/f, n = 15	N.A.	Awake post progressive training
**iso_12m**	12m	N = 0/1ⵜ+1** m/f, n = 3	N = 2/2 m/f, n = 12	Anesthesia
**awake_12m**	12m	N = 2/2 m/f, n = 12	N.A.	Awake post refresher training

*Also have WF-Ca^{2+} data
ⵜ mouse (and cohort) excluded from analyses (because single animal in that cohort)
**mouse excluded during analyses due to anatomical issues
m = months

Table S2. Censoring of functional data (fMRI)

Session	Mean Retained Frames	SD Retained Frames	Mean Censored Frames	SD Censored Frames
**iso_4m**	319.42	7.7881	15.577	7.7881
**iso_6m**	319.75	6.2433	15.25	6.2433
**awake_9m**	214.89	38.218	120.11	38.218
**iso_9m**	316.14	9.1394	18.857	9.1394
**awake_12m**	244.33	37.257	90.667	37.257
**iso_12m**	312.33	12.01	22.667	12.01

Supplementary Figures

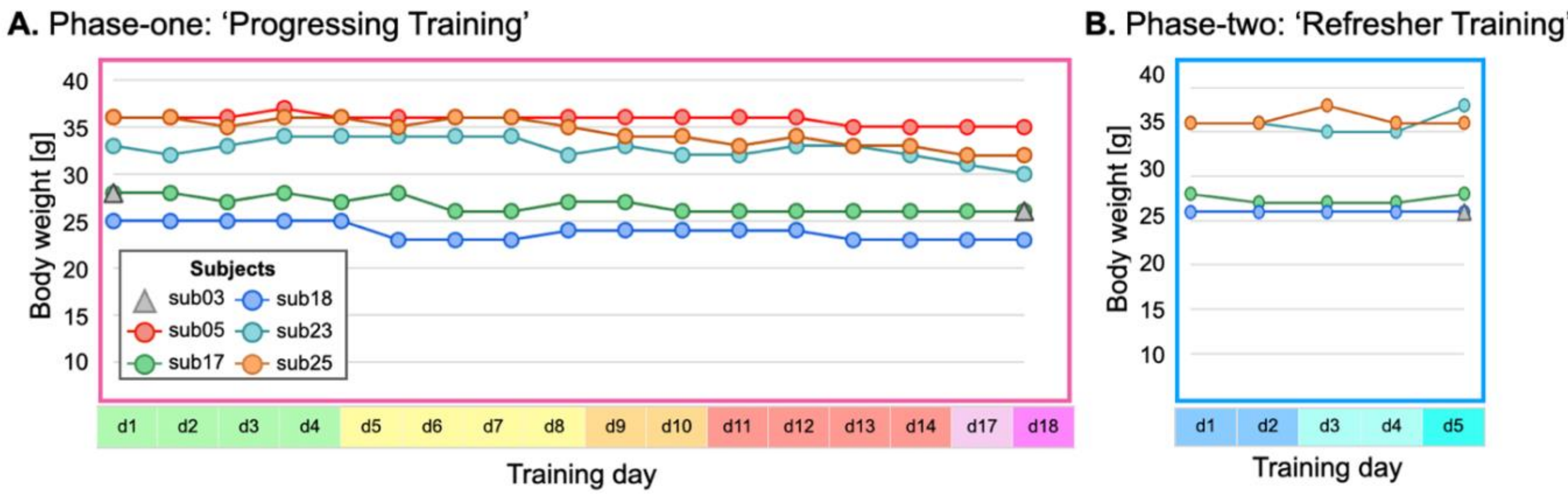

Fig. S1. Mouse body weight during biphasic acclimation training. A. Body weight measured daily during Phase one: 'Progressive training' for acclimating naïve mice to undergoing multimodal imaging whilst awake. A timeline for Phase one acclimation training is shown in **Fig. 1E**. Mice being acclimated are plotted in color. Body weight from a control mouse that did not undergo acclimation training is plotted in grey. **B.** As in (**A.**) for Phase two: 'Refresher training' for re-acclimating mice to undergoing multimodal imaging whilst awake. A timeline for Phase two acclimation training is shown in **Fig. 1G**.

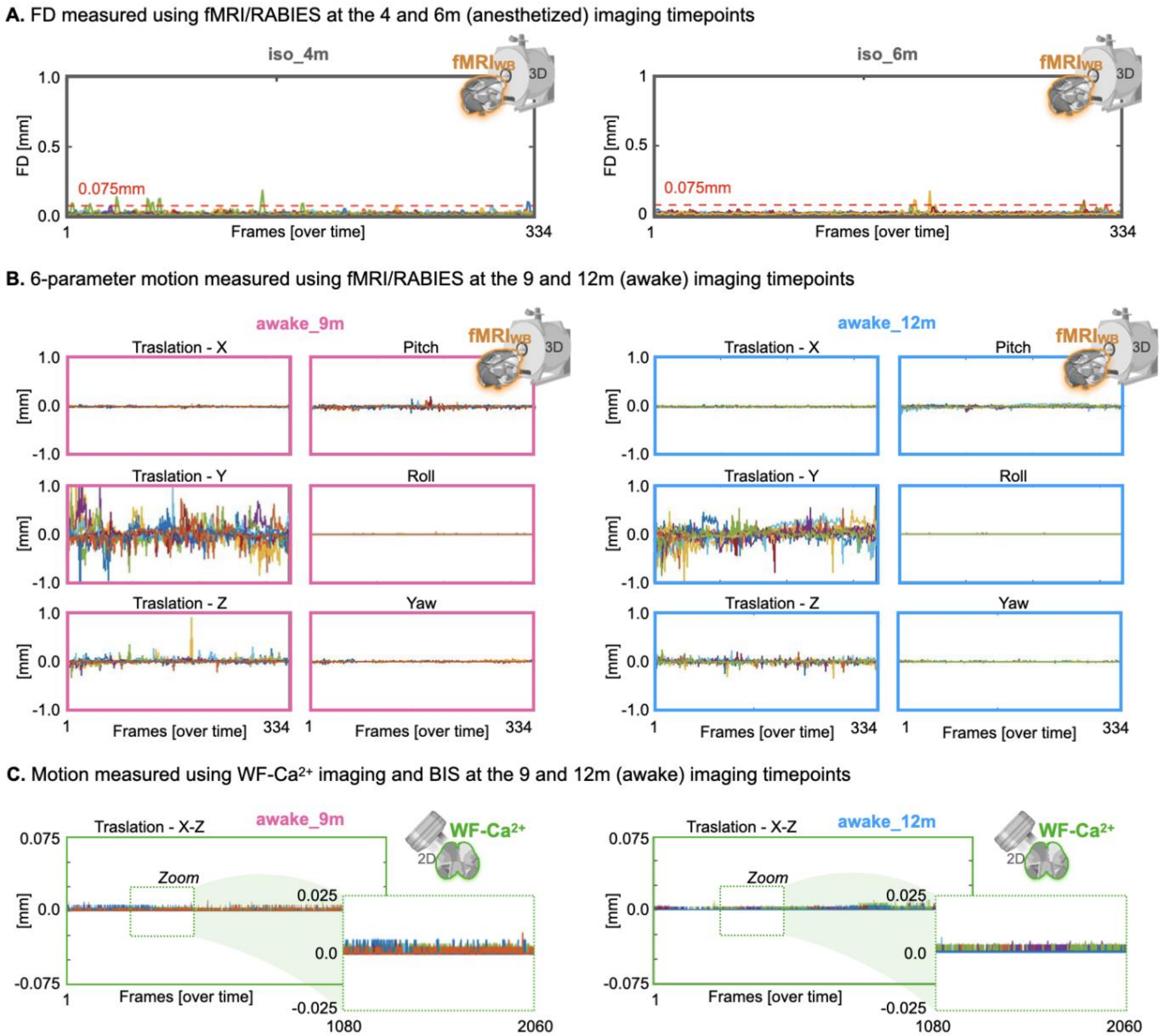

Fig. S2. Motion in multimodal imaging data. A. FD measured using fMRI data and RABIES the at 4 and 6m (anesthetized) imaging timepoints. Corresponding FD measures for the 9 and 12m anesthetized and awake imaging timepoints are shown in **Fig. 2B**. **B.** Motion from the 9 and 12m (awake) imaging timepoints broken down into 6-parameters. **C.** Motion in the WF-Ca^{2+} imaging data, estimated using BIS software, at the 9 and 12m (awake) imaging timepoints.

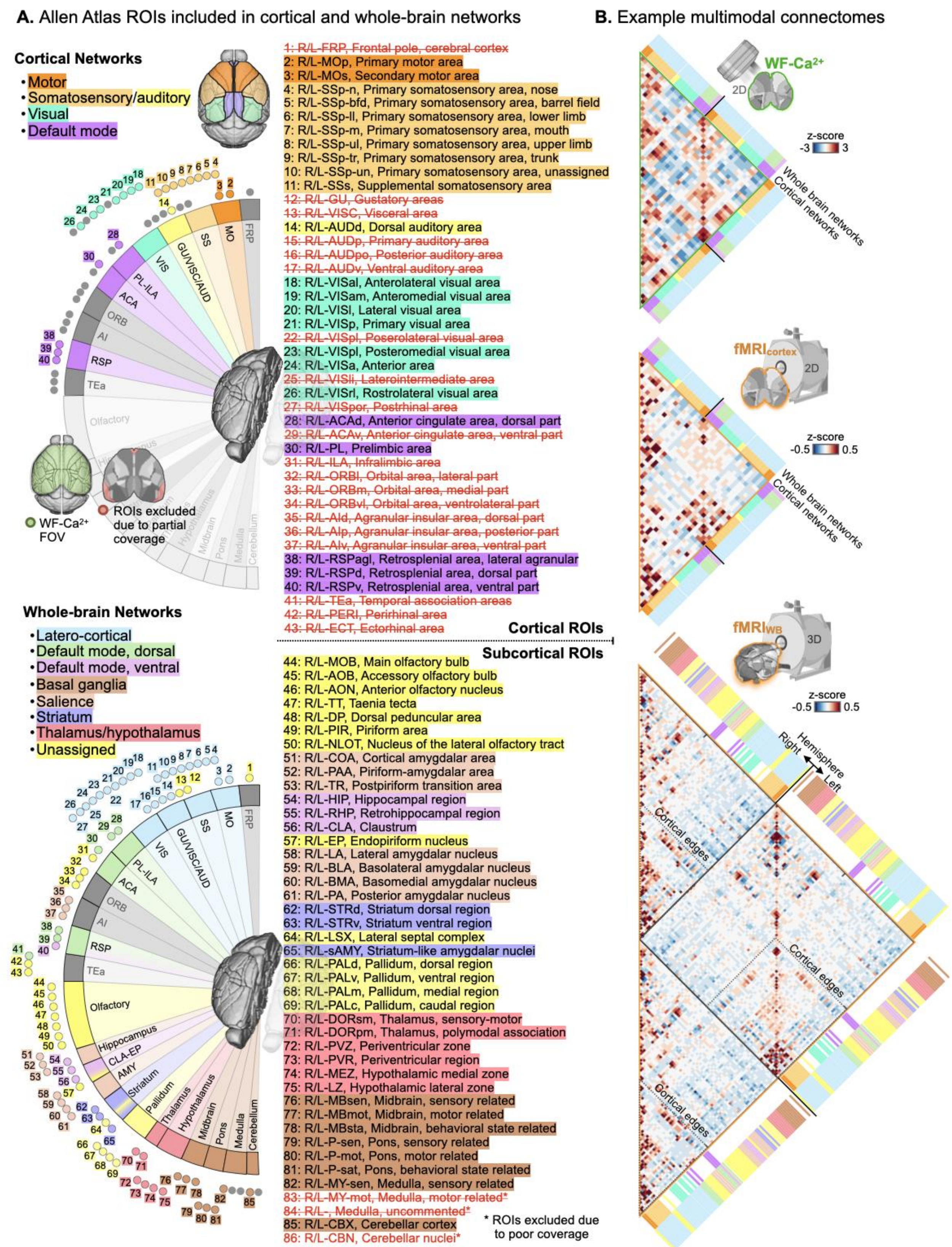

Fig. S3. Allen Atlas derived brain regions and a priori cortical and whole-brain networks. A. (top) Brain regions visible in the WF-Ca^{2+} imaging FOV used in all cortical network analyses. Regions were subdivided into four cortical networks. **(bottom)** Brain regions visible in fMRI data used in all whole-brain network analyses. Regions were subdivided into seven networks. **B.** Averaged connectomes from multimodal data including cortical (top) and whole-brain examples. Network assignments are color-coded.

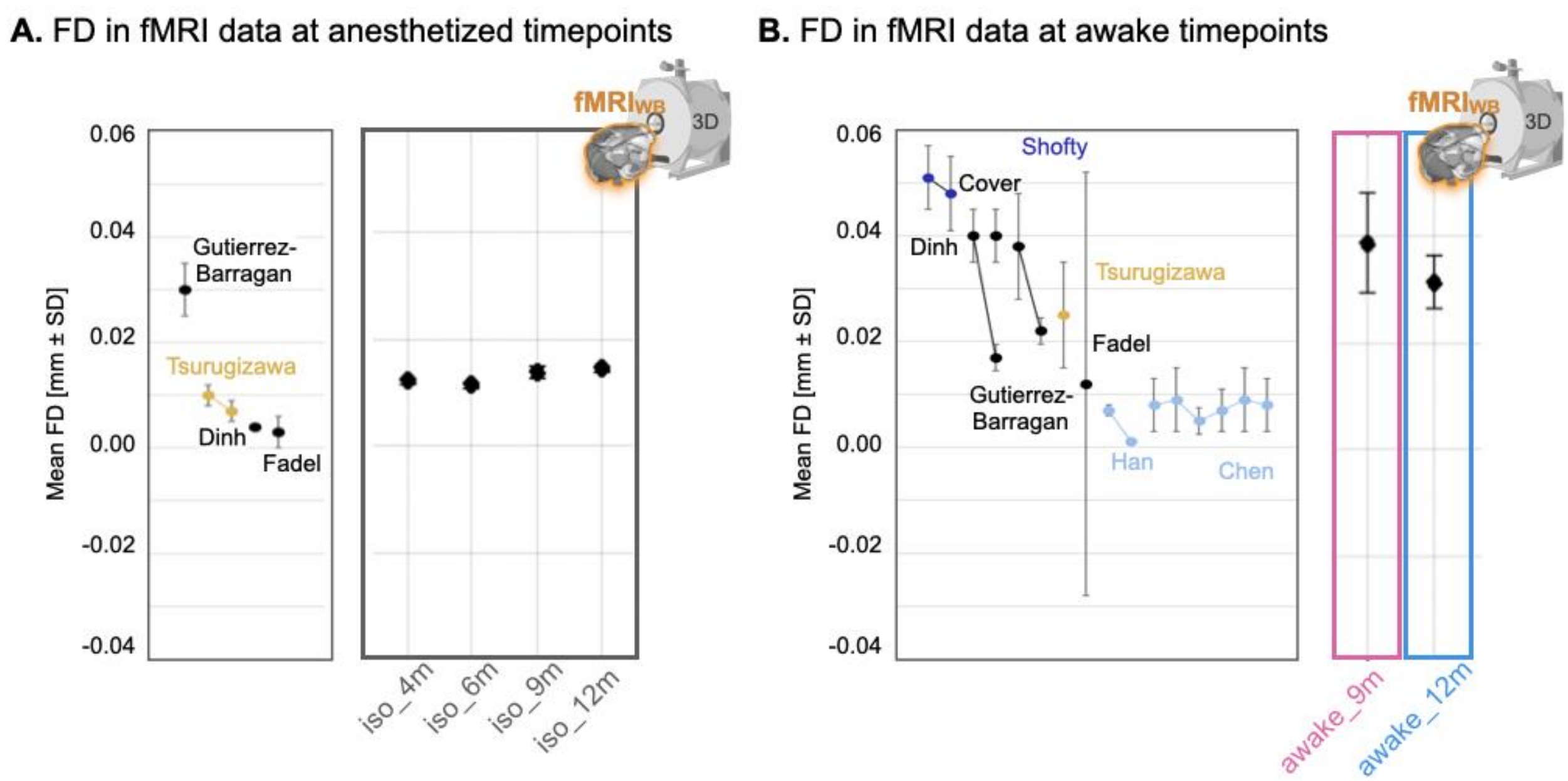

Fig. S4. FD in fMRI data estimated with RABIES compared to current literature. A,B: Left panels show reproduced values from **Figure S8** in a systematic review of awake mouse fMRI (Mandino *et al.*, 2024). Right panels show data from the current study. **A.** Mean FD in fMRI data acquired from anesthetized mice. **B.** Mean FD in fMRI data acquired from awake mice.

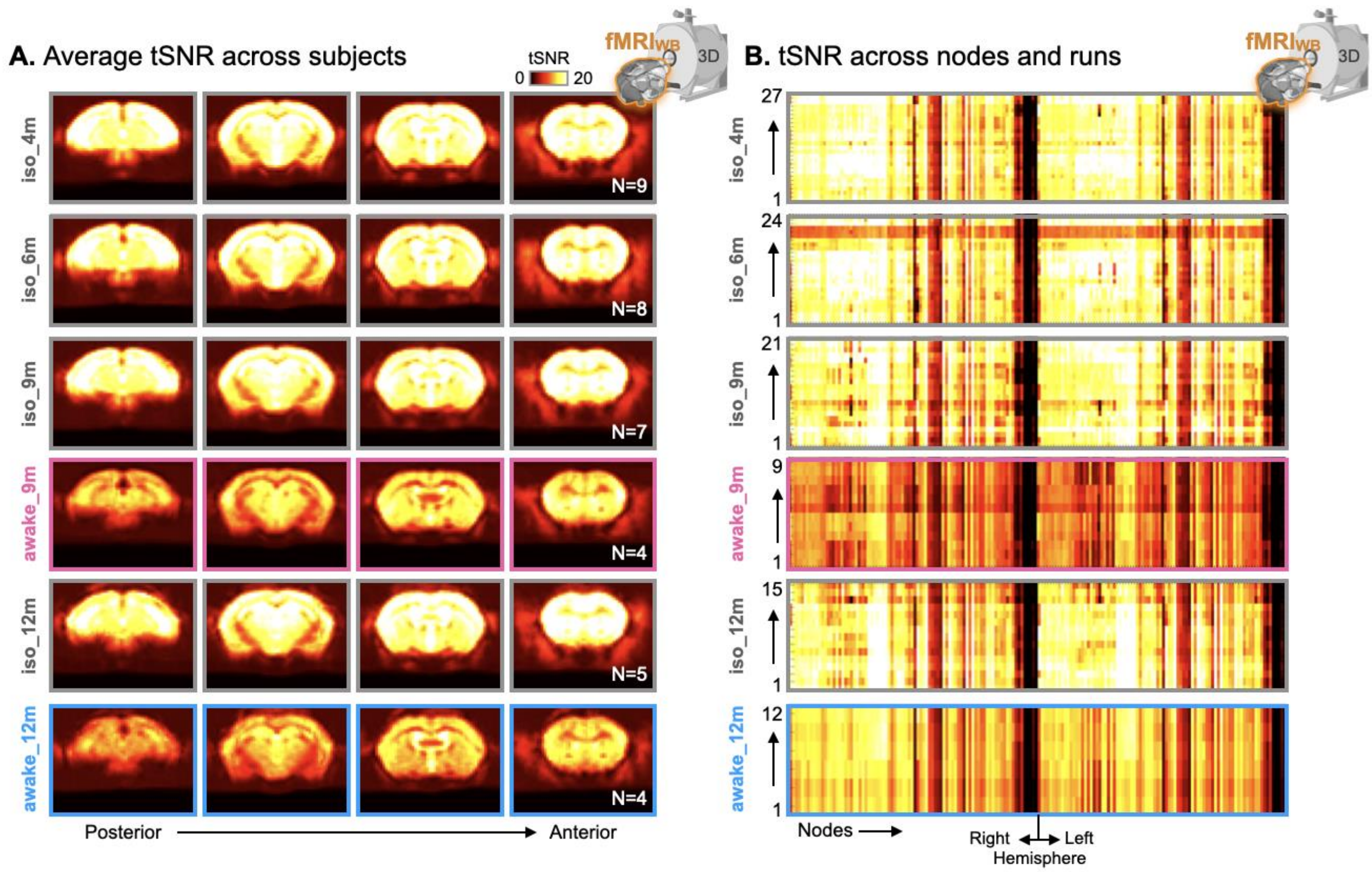

Fig. S5. Temporal SNR of fMRI data measured with RABIES. A. Example group averaged tSNR maps estimated with fMRI/RABIES at each imaging timepoint. **B.** Carpet plots output by RABIES which show tSNR estimated for each brain region (node), and run, at each imaging timepoint.

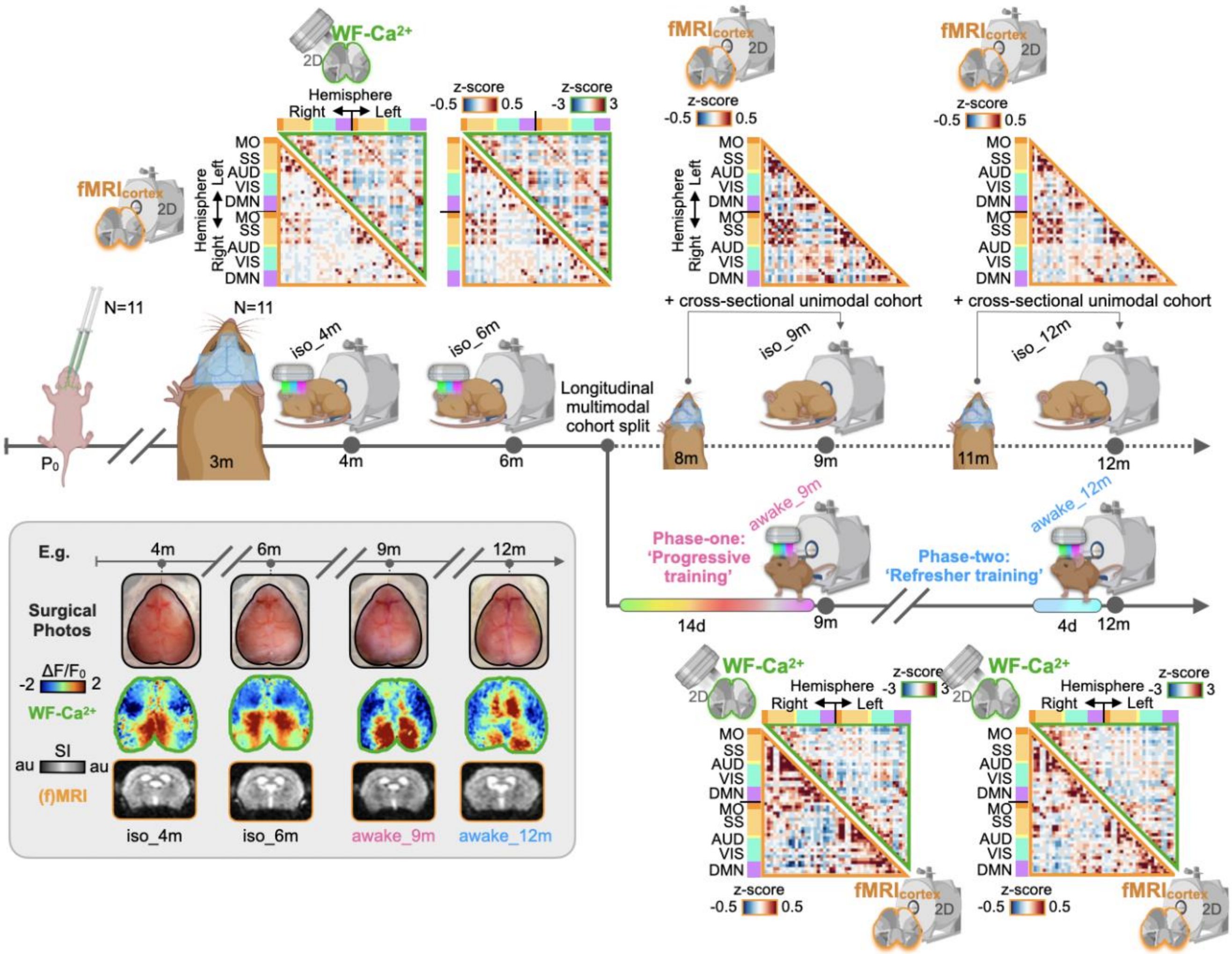

Fig. S6. Experimental timeline with example data. The timeline is copied from **Fig. 1A.** Above and below each multimodal imaging timepoint, averaged connectomes using WF-Ca^{2+} (green outline) or fMRI (orange outline) data from the cortex are shown. The inlay (grey background) shows images from an example mouse including photos of the head-plate surgery, as well as imaging frames from the WF-Ca^{2+} imaging and EPI timeseries.

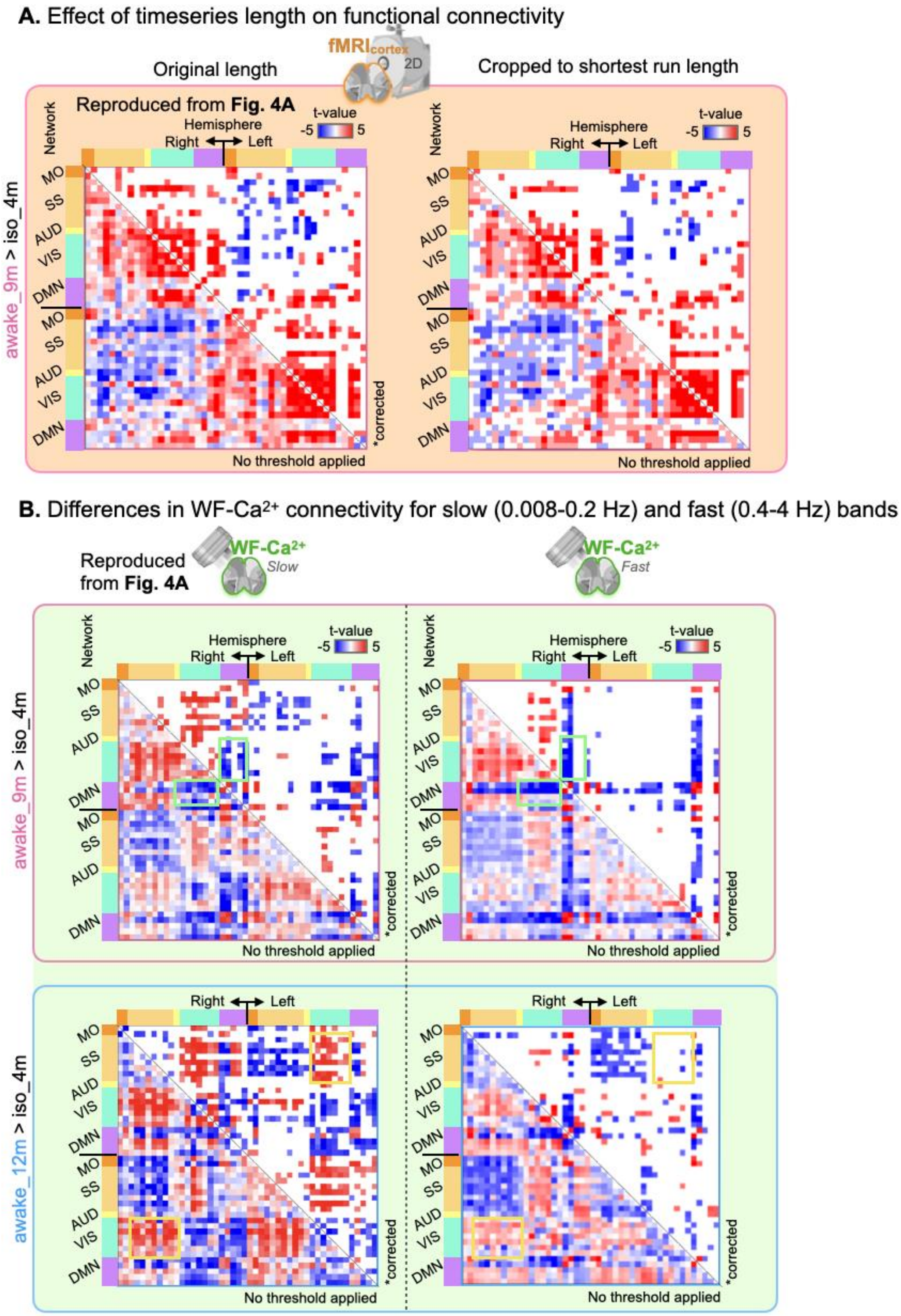

Fig. S7. Differences in connectivity between anaesthetized and awake mice: effects of timeseries lengths and frequency bands. A. Left: Reproduction of **Fig. 4A**, fMRI results. Right: Differences in connectivity between awake and anesthetized sessions with timeseries truncated to the shortest retained scan length across all datasets (example for awake_9m vs. iso_4m). **B.** Left: Reproduction of **Fig. 4A**, WF-Ca^{2+} results; Right: fast frequency band (0.4-4Hz) results.

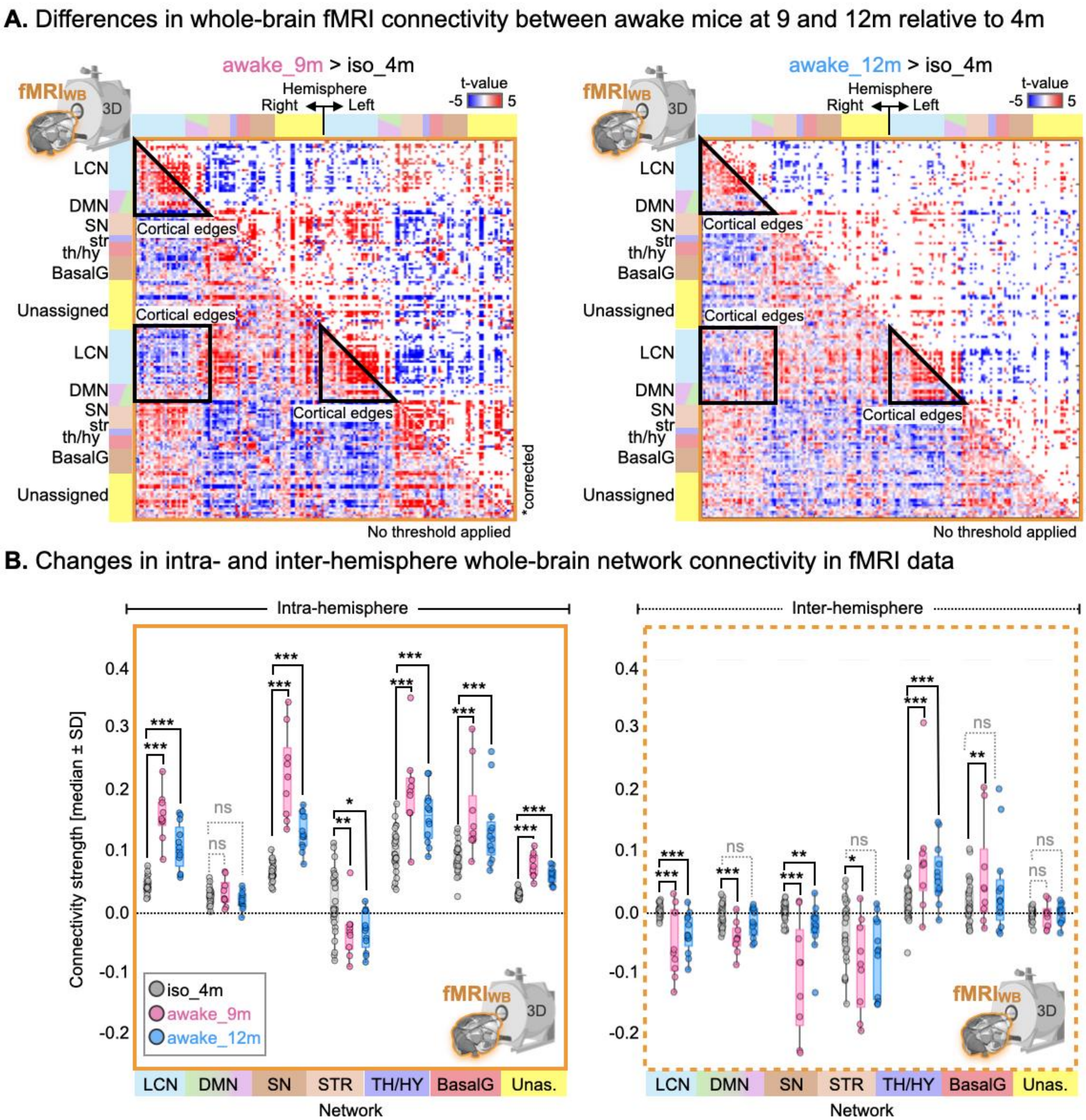

Fig. S8. Differences in whole-brain fMRI connectivity between anaesthetized and awake mice. As-in **Fig. 4A & C** with fMRI data from the whole-brain and seven *a priori* networks, **Fig. S3**. Edges within the cortex are indicated in (**A.**) **(**black outlines).

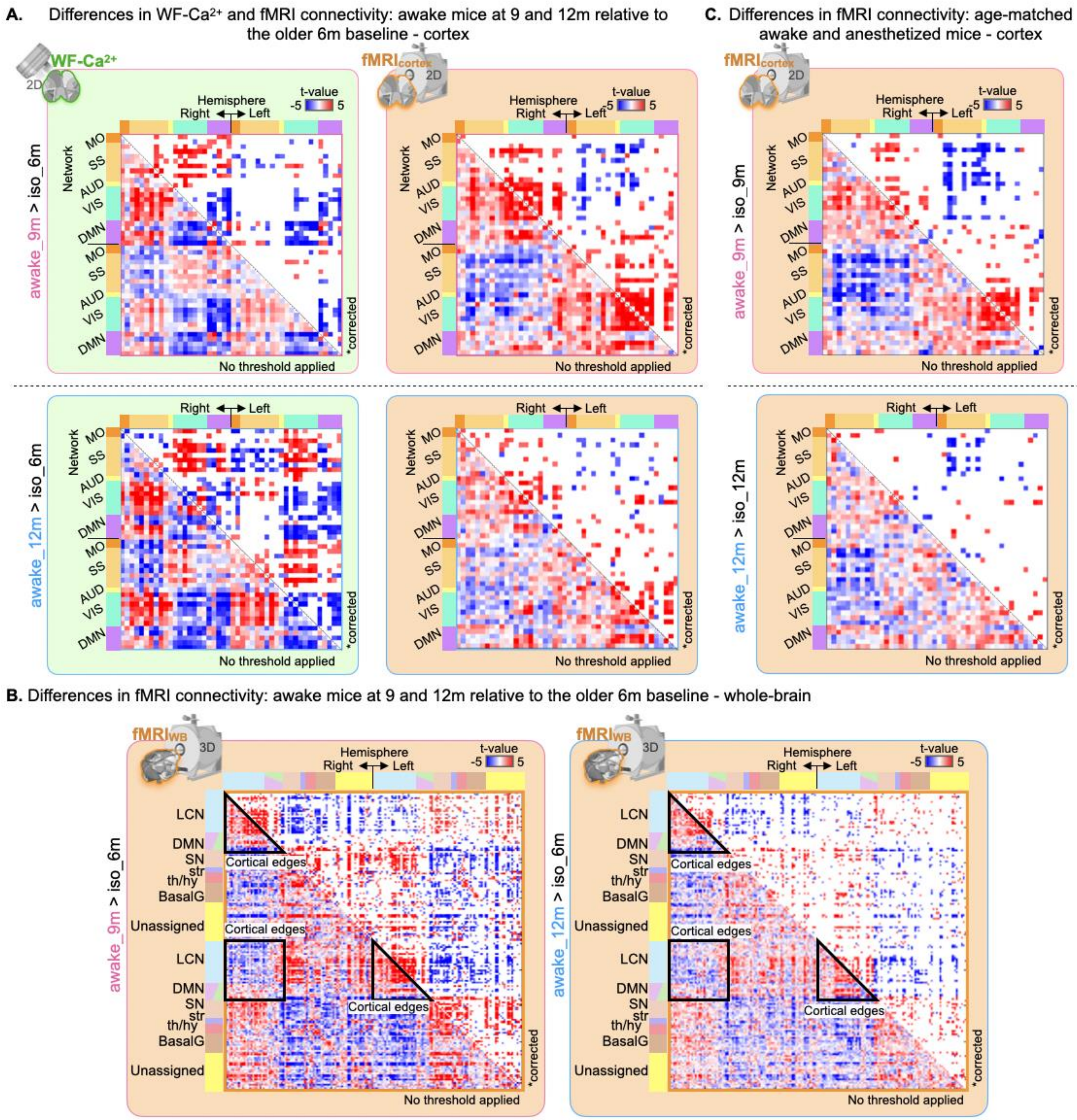

Fig. S9. Differences in functional connectivity between awake mice at 9 and 12m relative to other (anesthetized) baseline imaging timepoints. As-in **Fig. 3A** with data from the 6m (anesthetized) imaging timepoint used in-place of the 4m (anesthetized) imaging timepoint for cortical regions. Both WF-Ca^{2+} imaging data (green panels, left) and fMRI data (orange panels, right) are included. **B.** As-in (**A.**) for whole-brain fMRI data. **C.** As-in (**A.**) using the age-matched anesthetized imaging timepoint (fMRI data only).

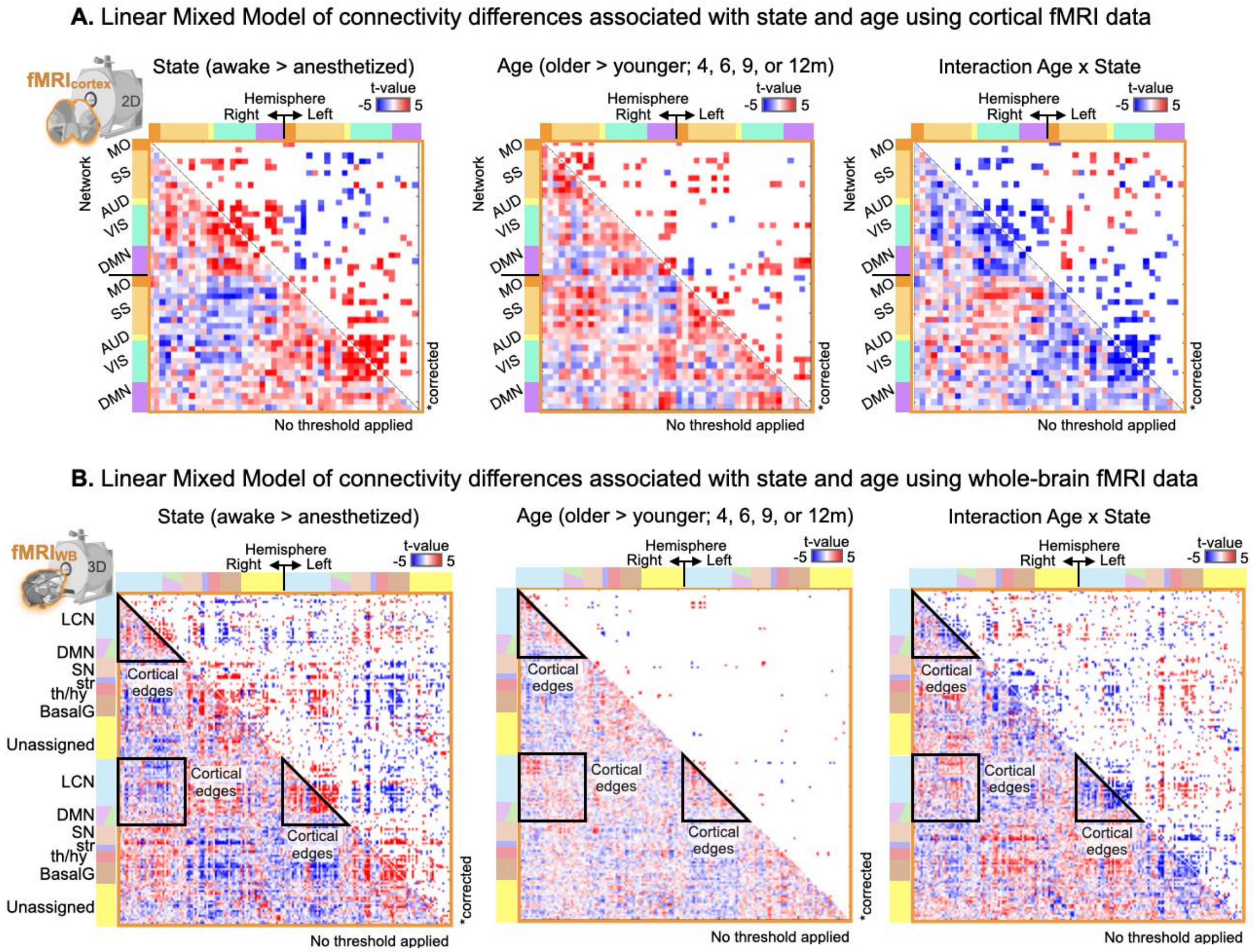

Fig. S10. Results from two Linear Mixed Models built using fMRI data to assess contributions of age. For each edge a linear mixed model tested the effects of State (awake versus anesthetized) and Age (4, 6, 9, or 12m) while accounting for animal identity as a random factor. The edges with significant effects (FDR-corrected, $q<0.05$) are displayed as t-value matrices for State (left), Age (middle) and their interaction (right). **A.** Model built using fMRI data from the cortex. **B.** Model built using fMRI data from the whole-brain. Edges within the cortex are indicated (black outlines).

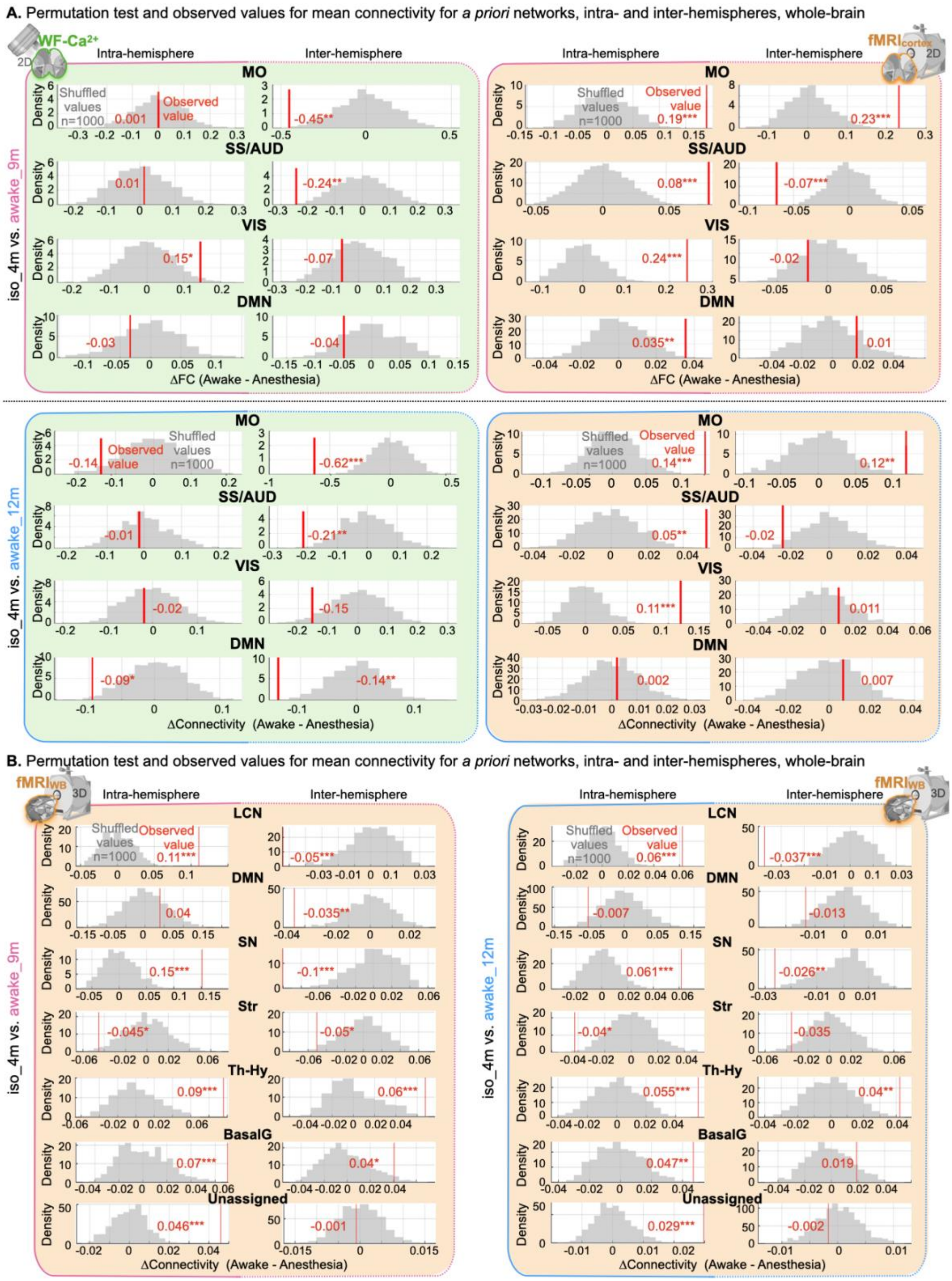

Fig. S11. Permutation test results for multimodal data and fMRI data from the whole-brain. For each network (rows) and hemisphere (columns: intra-hemisphere solid lines, inter-hemisphere dashed lines), histograms show mean connectivity strength differences generated from 1,000 permutations where awake and anesthetized labels were shuffled. ΔConnectivity plotted as t-values. **A.** Results from multimodal cortical data. **B.** Results from whole-brain fMRI data. Vertical red lines mark observed values from unshuffled data. Significance, corrected, displayed as: *p<0.05, **p<0.01, ***p<0.001).
